# Moutan Cortex Extract Modulates Macrophage Activation via Lipopolysaccharide-Induced Calcium Signaling and ER Stress-CHOP Pathway

**DOI:** 10.3390/ijms24032062

**Published:** 2023-01-20

**Authors:** Hyun-Ju Kim, Do-Hoon Kim, Wansu Park

**Affiliations:** 1 Department of Pathology, College of Korean Medicine, Gachon University, Seongnam 13120, Republic of Korea; 2 Department of Medical Classics and History, College of Korean Medicine, Gachon University, Seongnam 13120, Republic of Korea

**Keywords:** Moutan Cortex, *Paeonia suffruticosa*, macrophages, lipopolysaccharide, cytokine, CHOP, STAT3, nitric oxide, hydrogen peroxide

## Abstract

Moutan Cortex, *Paeonia suffruticosa* root, has long been used as a medicine for the treatment of inflammatory diseases. The aim of this study was to evaluate the modulative properties of Moutan Cortex water extract (CP) on endoplasmic reticulum (ER) stress-related macrophage activation via the calcium-CHOP pathway. RAW 264.7 mouse macrophages were activated by lipopolysaccharide (LPS), and the levels of various inflammatory mediators from RAW 264.7 were evaluated. The multiplex cytokine assay was used to investigate both cytokines and growth factors, and RT-PCR was used to investigate the expressions of inflammation-related genes, such as CHOP. Data represent the levels of NO and cytosolic calcium in LPS-stimulated RAW 264.7 were significantly inhibited by CP as well as hydrogen peroxide (*p* < 0.05). Minutely, NO production in LPS-stimulated RAW 264.7 incubated with CP at concentrations of 25, 50, 100, and 200 µg/mL for 24 h was 97.32 ± 1.55%, 95.86 ± 2.26%, 94.64 ± 1.83%, and 92.69 ± 2.31% of the control value (LPS only), respectively (*p* < 0.05). Calcium release in LPS-stimulated RAW 264.7 incubated with CP at concentrations of 25, 50, 100, and 200 µg/mL for 18 h was 95.78 ± 1.64%, 95.41 ± 1.14%, 94.54 ± 2.76%, and 90.89 ± 3.34% of the control value, respectively (*p* < 0.05). Hydrogen peroxide production in LPS-stimulated RAW 264.7 incubated with CP at concentrations of 25, 50, 100, and 200 µg/mL for 24 h was 79.15 ± 7.16%, 63.83 ± 4.03%, 46.27 ± 4.38%, and 40.66 ± 4.03% of the control value, respectively (*p* < 0.05). It is interesting that the production of IL-6, TNF-α, G-CSF, MIP-1α, MIP-2, and M-CSF in LPS-stimulated RAW 264.7 were significantly inhibited by CP (*p* < 0.05), while the production of LIX, LIF, RANTES, and MIP-1β showed a meaningful decrease. CP at concentrations of 25, 50, 100, and 200 µg/mL significantly reduced the transcription of *Chop*, *Camk2*α, *NOS*, *STAT1*, *STAT3*, *Ptgs2*, *Jak2*, *c-Jun*, *Fas*, *c-Fos*, *TLR3*, and *TLR9* in LPS-stimulated RAW 264.7 (*p* < 0.05). CP at concentrations of 25, 50, and 100 µg/mL significantly reduced the phosphorylation of STAT3, p38 MAPK, and IκB-α in LPS-stimulated RAW 264.7 (*p* < 0.05). These results suggest that CP might modulate macrophage activation via LPS-induced calcium signaling and the ER stress-CHOP pathway.

## 1. Introduction

Moutan Cortex is the cortex of *Paeonia suffruticosa* Andrews root (CP), which belongs to Paeoniaceae (family Ranunculaceae) [1]. The main ingredients of the cortex of *P. suffruticosa* have been reported to be paeonol, mudanpioside H, galloylpaeoniflorin, paeoniflorin, oxypaeoniflorin, gallic acid, and benzoylpaeoniflorin [2]. Moutan Cortex has traditionally been used in Asia to treat cardiovascular diseases, circulatory disorders, carbuncles, diabetes mellitus, appendicitis, and even autoimmune diseases [3,4,5]. However, there appear to have been no studies examining the activity of Moutan Cortex water extract (CP) on endoplasmic reticulum (ER) stress-related macrophage activation.

Macrophages, which are the main cells of the innate immune system, play a role in not only infectious diseases but also non-infectious diseases, such as atherosclerosis [6] and obesity-associated metabolic disorders related to their phagocytic activity and pattern recognition receptors activation [6,7,8]. Additionally, the unfolded protein response in ER-stressed macrophages is known to release calcium from ER via CHOP (GADD153) signaling [9,10]. It is interesting that the production of reactive oxygen species (ROS) in ER-stressed macrophages increases via CHOP signaling, resulting in the pyroptotic cell death (pyroptosis) of macrophages [11,12,13,14]. CHOP signaling in activated macrophages is also associated with the production of inflammatory factors such as nitrogen oxide (NO). For example, the excessive production of NO in endotoxins-activated macrophages promotes the rapid induction of CHOP via p38 mitogen-activated protein kinases (MAPK) signaling [15,16]. Moreover, since inflammatory reactions in endotoxins-stimulated macrophages are known to occur via I-kappa-B-alpha (IκB-α) signaling, it would also be beneficial to find medicinal substances that regulate IκB-α signaling in pyroptotic macrophages [17,18].

Although there have been reports of anti-inflammatory and antioxidant effects of Moutan Cortex, there have been no reports of calcium signaling related to ER stress and the action of the Moutan Cortex on CHOP activation in macrophages stimulated by lipopolysaccharide (LPS). Therefore, this study investigates the action of the Moutan Cortex on ER stress in LPS-stimulated macrophages and reports meaningful results.

## 2. Results

### 2.1. Extraction Yield and Total Flavonoid Content of CP

The hot water extract yield for CP is 20.67%. The total flavonoid content of CP is 6.85 mg RE/g extract.

### 2.2. Cell Viability

When CP was treated with 25, 50, 100, and 200 µg/mL in RAW 264.7 for 24 h, the resulting cell viability was 151.57 ± 12.61%, 151.29 ± 9.31%, 147.64 ± 7.32%, and 150.49 ± 4.23%, respectively, when only the media was treated, and there was no cytotoxicity (Table 1).

### 2.3. NO Level from RAW 264.7

The results of NO production in the group treated with LPS (1 µg/mL) only (LPS) were compared with those of the groups treated with LPS and CP. CP significantly inhibited LPS-induced NO production in RAW 264.7 (Table 2). The NO production of CP25 was 97.32 ± 1.55% compared to LPS, 95.86 ± 2.26% of CP50, 94.64 ± 1.83% of CP100, and 92.69 ± 2.31% of CP200, respectively. These results suggest that CP might modulate the ER stress signaling mediated by excessive NO production in activated macrophages.

### 2.4. Cytosolic Calcium Level

The results of cytosolic calcium levels in the group treated with LPS and the group treated with LPS and CP were compared. The calcium release of CP25 was found to be 95.78 ± 1.64% compared to LPS, 95.41 ± 1.14% of CP50, 94.54 ± 2.76% of CP100, and 90.89 ± 3.34% of CP200, respectively (Table 2). These results might be interpreted to mean that CP could regulate calcium signaling concerned with ER stress in the activated macrophages.

### 2.5. Hydrogen Peroxide Level in RAW 264.7

The results of hydrogen peroxide production in the group treated with LPS and the group treated with LPS and CP were compared. The level of hydrogen peroxide in RAW 264.7 was measured with the dihydrorhodamine 123 (DHR) assay. Finally, CP exerted inhibitory effects on hydrogen peroxide production in RAW 264.7 for 24, 48, and 72 h treatment in a dose-dependent manner (Table 2). The results of the DHR123 assay for 24 h treatment show that the hydrogen peroxide production of CP25 was 79.15 ± 7.16% compared to LPS, 63.83 ± 4.03% of CP50, 46.27 ± 4.38% of CP100, and 40.66 ± 4.03% of CP200, respectively. For 48 h treatment, the results were 80.81 ± 7.44% compared to LPS, 65.68 ± 3.16% of CP50, 48.84 ± 4.38% of CP100, and 42.63 ± 3.87% of CP200, respectively. For 72 h treatment, the results were 83.73 ± 7.84% compared to LPS, 69.95 ± 3.46% of CP50, 52.4 ± 4% of CP100, and 45.45 ± 4.08% of CP200, respectively. Our data indicate that CP might alleviate oxidative stress in macrophages and control the progression of ER stress cascade by reducing ROS production caused by endotoxin stimulation.

### 2.6. Cytokines Level

Macrophages produce various types of cytokines and growth factors through the stimulation of infectious pathogens such as endotoxins or by non-folded protein reactions caused by free cholesterol, thus causing inflammatory reactions. To investigate the activity of CP on macrophage activation accompanied by the massive production of cytokines, the effect of CP on cytokine production was simultaneously analyzed through in vitro experiments using RAW 264.7 cell culture supernatant, which was obtained after 24 h treatment with LPS and CP. The Bio-plex 200 system was used to carry out multiplex cytokine assay. The cytokines and growth factors investigated in this experiment were tumor necrosis factor (TNF)-α (TNFSF2), interleukin (IL)-6 (IL-6; BSF2), IL-10, CCL2 (MCP-1), CCL3 (MIP-1α), CCL4 (MIP-1β), CCL5 (RANTES), chemokine ligand 2 (CXCL2; macrophage-inflammatory peptide-2; MIP-2), CXCL5 (LIX), CSF1 (M-CSF), CSF2 (GM-CSF), CSF3 (G-CSF), CDF (LIF), and VPF (VEGF) (Table 2). Among them, CP significantly inhibited the production of IL-6, TNF-α, CSF3, CCL3, CXCL2, CXCL5, CCL4, CCL5, and CDF from endotoxins-activated RAW 264.7. The meaningful experimental results can be summarized as follows: CP at concentrations of 25, 50, 100, and 200 µg/mL significantly inhibited the production of IL-6, TNF-α, CSF3, CCL3, and CXCL2. CP at concentrations of 50, 100, and 200 µg/mL significantly inhibited the production of CXCL5. CP at concentrations of 25, 100, and 200 µg/mL significantly inhibited the production of CCL4. CP at concentrations of 25 and 50 µg/mL significantly inhibited the production of CCL5 and CDF. CP at concentrations of 25 and 200 µg/mL significantly inhibited the production of CSF1. CP at the concentration of 200 µg/mL significantly inhibited the production of CSF2. However, CP did not show any significant changes in the production of CCL2 and IL-10. The results suggest that CP leads to anti-inflammatory efficacy and macrophage activation by inhibiting the production of cytokines in macrophages stimulated by endotoxins.

### 2.7. Transcript Level of Inflammatory Gene Related to ER Stress

To examine the effect of CP on ER stress in further detail, the effect on the expression of genes related to ER stress—such as *Chop*, *Camk2α*, *Stat1*, *Stat3*, *Nos2*, and *Fas*—was checked. Further, infectious pathogens such as endotoxins cause macrophage activation, and inflammatory reactions caused by activated macrophages involve increased expression of inflammatory-related genes such as *Ptgs2*, *Jak2*, *c-Jun*, *c-Fos,* and *TLR9*. In view of these points, we investigated the mRNA expressions of inflammatory genes related to ER stress in macrophages using real-time PCR assay. In this study, CP at concentrations of 25, 50, 100, and 200 µg/mL significantly decreased the transcript levels of the *Chop*, *Camk2α*, *Stat1*, *Stat3*, *Nos2*, *Ptgs2*, *Jak2*, *Fas*, *c-Jun*, *c-Fos*, *TLR3*, and *TLR9* genes in RAW 264.7 stimulated by endotoxins (Table 2). The results suggest that CP might modulate macrophage activation by inhibiting the transcriptional levels of ER stress-related inflammatory genes such as *Chop* and *Camk2α*.

### 2.8. Activation of STAT3, p38 MAPK, and IkB-α

Since the activations and inflammatory responses of macrophages are related to the activations of STAT, p38 MAPK, and IκB-α, the effect of CP on the activations of STAT3, p38 MAPK, and IκB-α was investigated using flow cytometry assay. The results show that the phosphorylation levels of STAT3 were respectively decreased to 66.09 ± 0.34%, 68.42 ± 1.72%, and 63.03 ± 0.42% compared to that treated with LPS alone (Table 3). The phosphorylation levels of p38 MAPK in RAW 264.7 macrophages treated with CP at 25, 50, and 100 µg/mL were respectively decreased to 58.68 ± 1.1%, 56.75 ± 7.8%, and 56.84 ± 2.17% compared to that treated with LPS alone (Table 3). The phosphorylation levels of IκB-α were respectively decreased to 43.99 ± 0.03%, 41.64 ± 3.12%, and 41.34 ± 11.96% of that treated with LPS alone (Table 3). Taken together, the results indicate that CP might modulate macrophage activation related to ER stress by decreasing the activation of STAT3, p38 MAPK, and IκB-α.

## 3. Discussion

Various studies have been conducted to examine the ability of natural products with antioxidant and anti-inflammatory effects to relieve and treat symptoms of atherosclerosis [19]. Moutan Cortex has traditionally been used in Asia to treat cardiovascular diseases, circulatory disorders, carbuncles, diabetes mellitus, appendicitis, and even autoimmune diseases [3,4,5]. In 2017, Wang et al. reported anti-tumor and hepatoprotective effects of Moutan Cortex in addition to neuroprotective and cardiovascular protective activity [3]. Fu et al. reported that the administration of Moutan Cortex decreased levels of cytokines such as IL-1β, IL-6, IL-10, and CXCL2 in bronchoalveolar lavage fluid of rats with LPS-induced acute lung injury (ALI) and improved leukocyte infiltration in the alveolar space [4]. In 2020, Chen et al. reported that the water extract of Moutan Cortex inhibited nuclear factor kappa-B (NF-κB) activation and decreased IL-6 and TNF-α production in human monocytic cell (THP-1) cells in a dose-dependent manner [5]. Bai et al. reported that dietary Moutan Cortex improved serum antioxidant capacity and mitigate intestinal inflammation via the NF-κB pathway, including through the inhibition of NF-κB mRNA expression [20]. Jang et al. reported that Moutan Cortex extract protects hepatocytes (HepG2 cells) from arachidonic acid and iron-mediated oxidative stress via AMP-activated protein kinase activation and liver kinase B1 phosphorylation [21]. In 2007, Jiang et al. reported that Moutan Cortex ethanol extract (MCEE) inhibited the scratching behavior and systemic anaphylactic shock induced by compound 48/80 in mice in a dose-dependent manner, indicating that MCEE might antagonize immediate allergic reactions related to atopic dermatitis [22]. Moutan Cortex also decreased histamine release from compound 48/80-induced mast cells [22]. It has been reported that paeonol, one of the important components of Moutan Cortex, has the effect of relieving anti-atherosclerosis activity [23,24]. However, few studies have investigated the effect of the Moutan Cortex on atherosclerosis, specifically the anti-atherosclerosis effect using macrophages.

In 2014, Kim et al. reported that Moutan Cortex relieved Parkinson’s disease-like motor symptoms caused by 1-methyl-4-phenyl-1,2,3,6-tetrahydropyridine in a mouse model of Parkinson’s disease via the inhibition of mitochondrial dysfunction, including cytochrome C release and mitochondria-mediated apoptosis [25]. These findings, which were obtained by Kim et al. [25], are particularly interesting because the apoptosis of macrophages accompanied by the release of cytochrome C plays an important role in atheromatous plaque formation and plaque necrosis in atherosclerosis [6,26]. In other words, the activation of macrophages caused by the source of infection can be a factor in the deterioration of cardiovascular diseases such as atherosclerosis and the formation of plaque necrosis (atheromatous plaque) [6]. The formation of atheromata, which is an important lesion of atherosclerosis, is intensified with macrophage apoptosis, which is mainly triggered by ER stress, including an unfolded protein response, and the formation of these atheromata is promoted by macrophage activation accompanied by the activation of pattern recognition receptor signaling caused by infectious substances, such as endotoxins [27,28]. Since CHOP, an ER stress effector, is aggravated in atherosclerosis by promoting macrophage apoptosis, controlling the CHOP expression is an important aspect of alleviating atherosclerosis, and it is important to identify non-toxic natural substances that have such effects. Our experimental results using macrophages activated by LPS indicate that, since Moutan Cortex significantly inhibited the transcription of *Chop* in RAW 264.7, atherosclerosis might be alleviated by Moutan Cortex inhibiting the formation of atheromatous plaque. However, in this study, we have not investigated the effect of the Moutan Cortex on cytochrome C release from endotoxin-activated macrophage mitochondria and the resulting mitochondrial-mediated apoptosis. In fact, calcium is a secondary messenger that exhibits various physiological activities, such as muscle contraction through the formation of cross-bridge formation, synchronization of neuronal excitability with appropriate neurotransmitter release, and a role in fertilization by maturing oocytes, but calcium signaling is an important mechanism of ER stress wherein calcium is released from ER store through the phospholipase C pathway, including the inositol 1,4,5-trisphosphate (IP3) receptor activation, and apoptosis is induced as a result [29,30,31]. Interestingly, CHOP-induced ER oxidase 1 alpha (ERO1α) activates IP3-induced calcium release from ER in macrophages, where ER stress—such as the unfolded protein response—occurs and consequently induces apoptosis [32]. Therefore, alleviating the macrophage inflammatory response caused by endotoxins and controlling the cytosolic calcium release signaling caused by ER stress can help regulate the deterioration of atherosclerosis caused by macrophage apoptosis and advanced atheromata. The results of the present study show that Moutan Cortex significantly inhibits the increase of cytosolic calcium in endotoxin-stimulated macrophages, thus resulting in the modulation of ER stress-related macrophage activation. This means Moutan Cortex might be involved in changes in calcium signaling associated with endotoxin-induced macrophage activation.

CHOP (GADD134), one of the transcription factors, is activated by p38 MAPK, which promotes the release of calcium from ER into the cytoplasm through the IP3 receptor; it also increases the expression of genes that move into the nucleus to promote apoptosis and decreases the expression of genes that suppress apoptosis [32]. In this study, Moutan Cortex significantly inhibited the phosphorylation of p38 MAPK in endotoxins-activated RAW 264.7, which means that Moutan Cortex can reduce the expression of CHOP and consequently relieve ER stress by inhibiting the activation of p38 MAPK. Nie et al. reported in 2019 that p38 MAPK and NF-κB signaling molecules were involved in macrophage activation caused by LPS challenge accompanying massive secretion of cytokines such as IL-6 and TNF-α (i.e., hypercytokinemia) [33]. Moreover, Fu et al. reported in 2012 that Moutan Cortex decreased the levels of cytokines such as IL-1β, IL-6, IL-10, and CXCL2 in the bronchoalveolar lavage fluid of rats with LPS-induced ALI and improved leukocyte infiltration in the alveolar space [4]. The experimental results show that Moutan Cortex relieves the hypercytokinemia phenomena in endotoxins-activated RAW 264.7 through the regulation of p38 MAPK activation and IκB-α phosphorylation. This inhibition of p38 MAPK activation and IκB-α phosphorylation in Moutan Cortex seems to result in inhibiting the secretion of inflammatory cytokines such as IL-6, TNF-α, colony-stimulating factor 1, colony-stimulating factor 3, and macrophage inflammatory proteins (MIPs), altogether indicating that Moutan Cortex might alleviate endotoxins-induced macrophage activation.

By promoting Fas activation, CHOP contributes to macrophage apoptosis caused by ER stress [34], and unlike general ‘non-inflammatory apoptosis’, the reaction of macrophages stimulated and activated by infections such as endotoxins is called ‘proinflammatory pyroptosis’, which is processed via the greater activation of p38 MAPK [35]. Namely, unlike a general ‘apoptosis’ of macrophages stressed by free cholesterol, the antimicrobial response of macrophages with inflammasome activation induced by endotoxins is a programmed cell death involving a pro-inflammatory reaction, and it is called ‘pyroptosis’ [13,14]. Macrophages, which are stimulated by endotoxins and undergo a pyroptosis process, produce large amounts of inflammatory factors such as NO and cytokines and cause oxidative stress through ROS such as hydrogen peroxide. Therefore, it can be said that endotoxins-stimulated macrophages cause atherosclerosis to worsen through pyroptosis rather than apoptosis. In other words, it is logical to think that natural products with anti-pyroptosis can alleviate arteriosclerosis.

PCR data show that Moutan Cortex significantly suppressed the mRNA expressions of *Fas* and *Chop* in RAW 264.7 stimulated by endotoxins. It is therefore reasonable to say that Moutan Cortex has anti-pyroptosis properties in activated macrophages. This study was limited by the fact that it did not experimentally investigate the production of Fas and CHOP proteins secreted by activated macrophages. According to an impressive study by Cazanave et al., which examined the cooperative activity between CHOP and activator protein 1 (AP-1), ER stress-mediated apoptosis (i.e., lipoapoptosis) is dependent on CHOP and AP-1, which cooperatively mediate the expression of the p53 upregulated modulator of apoptosis during lipoapoptosis [36]. In line with the report by Cazanave et al., our data indicate that Moutan Cortex significantly decreases the expressions of AP-1 family members *c-Jun* and *c-Fos* in endotoxins-activated RAW 264.7. Interestingly, Meares et al. reported in 2014 that ER stress promotes the production of IL-6 through the Jak-STAT pathway in the neuronal inflammatory response [37]. Our results showed that Moutan Cortex significantly inhibits the phosphorylation of the STAT3 and mRNA expressions of *Stat1*, *Stat3*, and *Jak2* in the activated RAW 264.7, thus indicating that Moutan Cortex might modulate ER stress-related macrophage activation via Jak-STAT3 signaling.

It is notable that endotoxins increase the production of ROS and proinflammatory cytokines, such as TNF-α in activated macrophages, as well as the TNF-α-induced unfolded protein response depending on ROS in murine cells [11]. Similar to ROS, reactive nitrogen species (RNS) such as NO not only remove the source of infection but also affect the cell itself, thus enhancing ER stress. In fact, Chun et al. reported in 2007 that the methanol extract of Moutan Cortex significantly inhibits the production of NO, prostaglandin E2, IL-6, TNF- α, and IL-1β, as well as transcriptions of *Nos2* and *Ptgs2* in LPS-challenged RAW 264.7 via IκB-α signaling [38]. The PCR data in this study showed that Moutan Cortex water extract also suppresses the production of NO and hydrogen peroxide and the mRNA expressions of *Nos2* and *Ptgs2* in activated RAW 264.7. These results indicate that Moutan Cortex could alleviate macrophage activation via IκB-α signaling, including the massive production of RNS/ROS from endotoxins-activated macrophages. It is well known that calcium signaling is an important mechanism of ER stress wherein calcium is released from the ER store through the IP3 receptor activation and phospholipase C pathway [29,30,31], which is related to CHOP-induced ERO1α signaling in ER stress-induced macrophage apoptosis [32]. Unfortunately, this study could not evaluate the effect of the Moutan Cortex on phospholipase C signaling and ERO1α activation in activated macrophages. If it is CHOP that promotes the release of calcium release from ER in ER-stressed macrophages, then CAMK2 is activated by the increase in cytosolic calcium to induce ROS production and pro-apoptotic STAT1 activation [6,12]. The inhibitory effect of the Moutan Cortex on CAMK2α transcript in endotoxins-activated RAW 264.7 was confirmed in this study through PCR experiments, but there was no experiment investigating the effect of the Moutan Cortex on CAMK2/STAT1 activation. The toll-like receptor (TLR) is a pattern receptor protein that is located on the surface of the immune cell, which is an important factor in the process wherein innate immune cells recognize pathogens (mainly microbes) and cause immuno-inflammatory responses, thus activating AP-1 and NF-κB through MAPK and IκB-α signaling and ultimately resulting in the production of inflammatory factors such as cytokines [39]. The results suggest that the inhibitory effect of CP on mRNA expressions of TLR3 and TLR9 in activated macrophages could be regarded as having an anti-inflammatory activity related to the modulation of the TLR signaling pathway. Meanwhile, MCEE is known to antagonize immediate allergic reactions related to atopic dermatitis and decrease histamine release accompanied by mast cell degranulation caused by compound 48/80, which suggests Moutan Cortex has anti-allergic properties [22]. However, this study has not been able to identify the anti-allergic activity of Moutan Cortex concerned with mast cell degranulation.

This study found that CP modulates macrophage activation caused by LPS via the calcium-CHOP pathway, but there are several limitations as follows. We have not investigated the effect of the Moutan Cortex on cytochrome C release from endotoxin-activated macrophage mitochondria and the resulting mitochondrial-mediated apoptosis. The effect of the Moutan Cortex on phospholipase C signaling and ERO1α activation in activated macrophages could not be examined. The production of Fas and CHOP proteins in LPS-stimulated macrophages was not checked. The answer to which ingredients in CP significantly inhibit macrophage activation raises the need for the next study. For clinical trials of CP in inflammatory diseases, it will be necessary to confirm the anti-inflammatory efficacy in in vivo experiments.

## 4. Materials and Methods

The materials and methods for this study are based on previous studies [40,41,42]. More details are described in the supplementary file.

### 4.1. Materials

Dulbecco’s modified Eagle medium, phosphate buffer saline, LPS, baicalein, and indomethacin were purchased from Millipore (Billerica, MA, USA).

### 4.2. Preparation of CP

Commercial Moutan Cortex was purchased from Omniherb (Daegu, Korea). Moutan Cortex (voucher specimen No. 21032) was authenticated by referring to the website of the Korean Ministry of Food and Drug Safety (KFDA) (https://www.nifds.go.kr/nhmi/analscase/snststMnl/view.do?selectedSnststMnlNo=134 (accessed on 5 March 2021) or https://www.nifds.go.kr/nhmi/hbdc/ofcmhbdc/view.do?selectedDmstcOfcmNo=161&selectedMdntfNo=266 (accessed on 5 March 2021)). Moutan Cortex materials were extracted using hot water [40,41,42].

### 4.3. The Total Flavonoid Content (TFC) of CP

The TFC of CP was determined using the diethylene glycol colorimetric assay with a TRIAD LT spectrofluorometer (Dynex, West Sussex, UK) at 405 nm [40,41,42].

### 4.4. Effect of CP on Cell Viability

Murine macrophage RAW 264.7 cell line (passage number 2) was obtained from the Korea Cell Line Bank (Seoul, Korea). Cell viability was measured using a modified MTT assay according to a previously described method with a TRIAD LT spectrofluorometer at 540 nm [40,41,42]. Briefly, after cells were stabilized on a 96-well plate, cell culture media or 25, 50, 100, and 200 µg/mL of CP were treated and the cell viability was measured 24 h later.

### 4.5. Effect of CP on Level of NO, Cytosolic Ca^2+^, and Hydrogen Peroxide

NO production was measured using Griess reagent assay [40,41,42]. After the cells were stabilized, LPS and/or CP were treated and cultured for 24 h. After the culture was completed, 100 uL of a grease reagent was added to 100 uL of the cell culture supernatant, and after 15 min, absorbance was measured at 540 nm with a TRIAD LT spectrofluorometer. NO production and absorbance are proportional.

Intracellular calcium level was measured with Fluo-4 calcium assay [40,41,42]. Briefly, after the cells were stabilized in a 96-well plate, LPS and/or CP were treated and incubated for 18 h. Then, the Fluo-4 reagent was treated at 37 °C for 30 min. After the incubation, fluorescence was measured using a TRIAD LT spectrofluorometer with excitation and emission filters of 485 and 535 nm. Cytosolic calcium levels and fluorescence are proportional.

The production of hydrogen peroxide in cells was measured with the dihydrorhodamine 123 Assay [40,41,42]. Briefly, after the cells were stabilized in a 96-well plate, a dihydrorhodamine 123 reagent was added to the cells and incubated for 1 h. Then, LPS and/or CP were treated and incubated for 24, 48, and 72 h. After incubation, fluorescence was measured using a TRIAD LT spectrofluorometer with excitation and emission filters of 485 and 535 nm. The higher the fluorescence, the higher the production of ROS.

### 4.6. Effect of CP on Cytokine Production

Multiplex Cytokine Assay kits of Millipore were used to evaluate the concentrations of cytokines in RAW 264.7 with a Bio-Plex 200 suspension array system (Bio-Rad, Hercules, CA, USA) [40,41,42]. Briefly, after the cells were stabilized, LPS and/or CP were treated and cultured for 24 h. After the culture was completed, 50 uL of the cell culture supernatant was added to the magnetic beads in a 96-Well Flat Bottom Plate included in the Assay kit. After shaking at room temperature for 60 min, magnetic beads were washed with Wash Buffer (1X) by the Hand-Held Magnetic Plate Washer. After washing the magnetic beads, 25 uL of Detection Antibody Mix was added to the magnetic beads. After washing the magnetic beads, 50 uL of Streptavidin-PE was added to the magnetic beads. After washing the magnetic beads, 120 uL of Reading Buffer was added to the magnetic beads and cytokine levels were analyzed with Bio-Plex 200 suspension array system.

### 4.7. Effect of CP on Transcript Level of Inflammatory Genes

The transcript levels of *Chop*, *Camk2α*, *Stat1*, *Stat3*, *Jak2*, *Fas*, *c-Jun*, *c-Fos*, *Nos2*, *Ptgs2*, *TLR3, TLR9,* and *β-Actin* were quantified with real-time PCR assay using the CFX96 Real-Time PCR Detection System (Bio-Rad) [40,41,42]. Briefly, at the end of 18 h incubation with LPS and/or CP, RAW 264.7 cells were lysed and total RNA was isolated using a NucleoSpin RNA kit (Macherey-Nagel, Duren, Germany). RNA quantity and quality were confirmed using the Experion RNA StdSens Analysis kit (Bio-Rad) and Experion Automatic Electrophoresis System (Bio-Rad). cDNA was synthesized from 1 µg total RNA using the iScript cDNA Synthesis kit (Bio-Rad). The reaction mix (20 μL) including RNA template (1 μg) was incubated in a thermal cycler (C1000 Thermal Cycler, Bio-Rad) according to the manufacturer’s protocol (priming at 25 °C for 5 min, reverse transcription at 46 °C for 20 min, and RT inactivation at 95 °C for 1 min). cDNA, iQ SYBR Green Supermix (Bio-Rad), and Forward/Reverse primers for each target gene were added to the wells of a qPCR plate. Then, Real-time PCR was performed using the following protocol: denaturation of DNA at 95 °C for 3 min, followed by 40 cycles of 95 °C for 10 s and 55 °C for 30 s. Relative changes in gene expression were calculated using the 2^−ΔΔCt^ cycle threshold method with *β-Actin*. The GenBank accession numbers used to design the primers are listed in Table 4.

### 4.8. Effect of CP on Phosphorylation of STAT3, p38 MAPK, and IκB-α

The effect of CP on the phosphorylation of STAT3, p38 MAPK, and IκB-α was measured by flow cytometry assay [40,41,42]. Briefly, RAW 264.7 macrophages were seeded in 6-well plates (1 × 10^6^ cells/well) and incubated with LPS and/or CP for 18 h. After incubation, cells were harvested and washed with Flow Cytometry Staining Buffer. Then, RAW 264.7 was stained with Fixable Viability Dye eFluor 520, phospho-STAT3 antibody, phospho-p38 MAPK antibody, phospho-IκB-α antibody, and mouse IgG2b kappa Isotype control antibody. The phosphorylation levels of STAT3, p38 MAPK, and IκB-α were analyzed using Attune NxT flow cytometer (Thermo Fisher Scientific, Waltham, MA, USA) with Attune NxT software version 2.6 (Thermo Fisher Scientific). A serial gating strategy used forward scatter versus side scatter plots, forward scatter versus viability stain plots, and the target antibody expression plots. Unstained cells were used as the negative controls for gating. Details regarding the operation of the Attune can be found in the Attune User Guide (https://assets.thermofisher.com/TFS-Assets/LSG/manuals/100024235_AtuneNxT_HW_UG.pdf (accessed on 10 May 2021)).

### 4.9. Statistical Analyses

Data are representative of at least three independent experiments. The distribution normality of experimental data was analyzed with GraphPad Prism 4.0 (GraphPad Software, San Diego, CA, USA). Data are normally distributed and values are expressed in means ± standard deviation. Statistical differences between groups were assessed by ANOVA and Tukey post hoc test with GraphPad Prism 4.0.

## 5. Conclusions

Through this experimental study, the following points were revealed. First of all, the water extract yield for CP is 20.67%, and the total flavonoid content of CP is 6.85 mg RE/g extract. CP at concentrations of 25, 50, 100, and 200 µg/mL significantly inhibited NO production, calcium release, hydrogen peroxide production, and the secretion of cytokines (i.e., IL-6, TNF-α, G-CSF, MIP-1α, MIP-2, and M-CSF) in LPS-stimulated RAW 264.7 (*p* < 0.05) as well as mRNA expressions of *Chop*, *Camk2α*, *NOS*, *STAT1*, *STAT3*, *Ptgs2*, *Jak2*, *c-Jun*, *Fas*, *c-Fos*, *TLR3*, and *TLR9* (*p* < 0.05), which means that the mitigation effect of CP on macrophage activation was through calcium-related CHOP pathway. Additionally, CP at concentrations of 25, 50, and 100 µg/mL significantly reduced the phosphorylation of STAT3, p38 MAPK, and IκB-α in LPS-stimulated RAW 264.7 (*p* < 0.05). These results suggest that CP might modulate macrophage activation via LPS-induced calcium signaling and the ER stress-CHOP pathway.

## Figures and Tables

**Table 1:** Effects of Moutan Cortex water extract (CP) on cell viabilities of RAW 264.7.

Treatment Group	Cell Viability (%)		
Basal (media only)	100.00	±	4.52
25 µg/mL of CP	151.57	±	12.61 ^#^
50 µg/mL of CP	151.29	±	9.31 ^#^
100 µg/mL of CP	147.64	±	7.32 ^#^
200 µg/mL of CP	150.49	±	4.23 ^#^

Values are the mean ± SD of three independent experiments. ^#^, *p* < 0.05 vs. Basal (media only). Statistical significances were checked with ANOVA and Tukey test.

**Table 2:** Effects of Moutan Cortex water extract (CP) on lipopolysaccharide (LPS)-activated RAW 264.7.

Inflammatory Factor	Basal (Media Only)			LPS (LPS Alone)			Concentration (µg/mL) of CP with Lipopolysaccharide (1 µg/mL)											
							25			50			100			200		
Nitric Oxide (%)	100.00	±	2.92	201.98	±	3.55	197.49	±	2.94 **	194.58	±	3.94 ***	192.21	±	4.00 ***	188.64	±	4.44 ***
Cytosolic Calcium (%)	100.00	±	1.90	104.49	±	2.32	100.08	±	1.71*	99.69	±	1.19 *	98.79	±	2.88 **	94.97	±	3.49 ***
Hydrogen Peroxide (24 h)	100.00	±	7.23	212.26	±	21.74	168.01	±	15.19 ***	135.49	±	8.55 ***	98.20	±	9.30 ***	86.31	±	8.55 ***
Hydrogen Peroxide (48 h)	100.00	±	7.71	202.68	±	19.97	163.80	±	15.09 ***	133.12	±	6.41 ***	98.99	±	8.88 ***	86.41	±	7.84 ***
Hydrogen Peroxide (72 h)	100.00	±	8.88	212.74	±	25.08	178.12	±	16.68 **	148.81	±	7.36 ***	111.47	±	8.52 ***	96.68	±	8.68 ***
IL-6 (pg/mL)	40.00	±	3.28	27,159.00	±	230.53	25,932.00	±	123.79 **	25,848.25	±	457.21 *	25,431.25	±	694.80 *	25,622.67	±	170.93 **
MCP-1 (pg/mL)	38.83	±	6.71	2951.50	±	327.74	2608.50	±	241.64	2556.17	±	224.95	2387.33	±	332.41	2417.00	±	227.05
TNF-α (pg/mL)	201.17	±	43.48	6647.88	±	97.18	5642.83	±	114.48 **	5952.38	±	370.63 **	5729.13	±	583.20 *	6047.33	±	93.11 *
G-CSF (pg/mL)	175.67	±	44.88	27,526.50	±	150.23	25,875.50	±	93.66 **	26,044.67	±	261.50 **	26,004.00	±	534.14 *	25,951.00	±	51.80 ***
GM-CSF (pg/mL)	34.00	±	7.21	17,425.00	±	306.59	16083.67	±	1145.00	15,891.67	±	2463.69	13095.17	±	1767.27	15,996.83	±	841.74 *
IL-10 (pg/mL)	25.67	±	3.79	4529.33	±	627.70	4674.00	±	260.85	4829.33	±	338.25	4659.83	±	831.75	4841.67	±	165.21
LIF (pg/mL)	38.50	±	3.19	8497.50	±	289.28	7748.75	±	437.68*	7859.63	±	250.35 *	7633.50	±	1278.55	8127.13	±	473.55
LIX (pg/mL)	554.25	±	35.74	6535.25	±	243.25	5992.83	±	159.99	5966.83	±	286.08 *	5842.75	±	444.35 *	6108.50	±	163.19 *
M-CSF (pg/mL)	33.50	±	0.50	39.33	±	3.40	31.63	±	3.35*	31.00	±	1.80 ***	33.67	±	2.25 ***	33.75	±	2.53 *
MIP-1α (pg/mL)	3954.63	±	1365.10	27,064.38	±	108.21	25,348.00	±	159.69 **	25,312.00	±	588.39*	25,248.75	±	394.20 **	25,528.17	±	142.82 **
MIP-1β (pg/mL)	2938.33	±	207.54	22,371.33	±	347.30	20,600.13	±	502.24*	21,125.63	±	1433.70	20,115.33	±	507.80 **	20,583.63	±	380.70 **
MIP-2 (pg/mL)	62.50	±	17.06	23,431.75	±	383.83	21,235.17	±	135.30 **	22,297.33	±	706.70 **	21,889.83	±	1104.75 *	22,200.50	±	680.97 *
RANTES (pg/mL)	64.17	±	11.45	11,415.50	±	738.99	9406.67	±	312.65*	9832.33	±	836.91 *	9174.00	±	829.30	10,096.00	±	306.18
VEGF (pg/mL)	187.75	±	32.95	3439.63	±	322.20	3248.13	±	302.35	3373.88	±	186.46	3319.13	±	570.31	3523.88	±	506.10
*Chop* mRNA (ratio)	1.00	±	0.42	27.09	±	8.63	1.38	±	0.12 **	0.77	±	0.13 **	0.75	±	0.32 **	1.75	±	1.35 **
*Camk2*α mRNA (ratio)	1.01	±	0.32	9.26	±	2.72	2.91	±	0.37 **	1.97	±	0.11 **	2.32	±	1.25 **	3.61	±	1.51 *
*Stat1* mRNA (ratio)	1.00	±	0.30	4.26	±	0.06	0.76	±	0.14 ***	0.65	±	0.09 ***	0.63	±	0.13 ***	1.38	±	0.23 ***
*Stat3* mRNA (ratio)	1.01	±	0.31	3.36	±	0.24	1.20	±	0.41 ***	1.20	±	0.59 ***	1.18	±	0.46 ***	1.16	±	0.18 ***
*Jak2* mRNA (ratio)	1.00	±	0.39	4.90	±	0.92	0.72	±	0.10 ***	0.33	±	0.01 ***	0.32	±	0.02 ***	0.92	±	0.10 ***
*Fas* mRNA (ratio)	1.02	±	0.18	51.52	±	6.90	32.31	±	6.04*	14.63	±	0.29	29.89	±	6.34 **	21.44	±	4.85 **
*c-Jun* mRNA (ratio)	1.02	±	0.07	18.90	±	1.71	1.96	±	1.28 ***	1.64	±	0.81 ***	1.39	±	0.70 ***	0.88	±	0.51 ***
*c-Fos* mRNA (ratio)	1.04	±	0.13	48.73	±	1.46	20.23	±	3.05 ***	10.51	±	1.25 ***	16.67	±	0.29 ***	27.88	±	3.23 ***
*Nos2* mRNA (ratio)	1.00	±	0.07	150.27	±	4.90	75.71	±	14.94 **	30.33	±	7.73 ***	84.76	±	4.29 ***	75.68	±	3.51 ***
*Ptgs2* mRNA (ratio)	1.00	±	0.03	1509.93	±	34.75	515.01	±	55.58 ***	168.16	±	13.40 ***	520.00	±	68.01 ***	643.10	±	53.53 ***
*TLR3* mRNA (ratio)	1.02	±	0.11	5.30	±	0.22	1.61	±	0.05 ***	1.29	±	0.10 ***	1.44	±	0.20 ***	3.43	±	0.27 **
*TLR9* mRNA (ratio)	1.01	±	0.35	5.35	±	0.44	0.86	±	0.07 ***	0.82	±	0.04 ***	0.99	±	0.15 ***	1.76	±	0.26 ***

Values are the mean ± SD (*n* = 4). *, *p* < 0.05 vs. LPS; **, *p* < 0.01 vs. LPS; ***, *p* < 0.001 vs. LPS. Statistical significances were checked with ANOVA and Tukey test.

**Table 3:** Effects of Moutan Cortex water extract (CP) on levels of phosphorylated STAT3, phosphorylated p38 MAPK, and phosphorylated IκB-α in lipopolysaccharide (LPS, 1 µg/mL)-activated RAW 264.7.

Treatment Group	Phosphorylation Level (%)								
	STAT3			p38 MAPK			IκB-α		
LPS (1 µg/mL) only	100.00	±	0.61	100.00	±	2.92	100.00	±	2.93
LPS + 25 µg/mL of CP	66.09	±	0.34 ***	58.68	±	1.10 ***	43.99	±	0.03 ***
LPS + 50 µg/mL of CP	68.42	±	1.72 ***	56.75	±	7.80 **	41.64	±	3.12 ***
LPS + 100 µg/mL of CP	63.03	±	0.42 ***	56.84	±	2.17 ***	41.34	±	11.96 **
LPS + Baicalein (25 µM)	64.24	±	1.03	51.66	±	0.94	51.70	±	2.22

Values are the mean ± SD of three independent experiments. ** *p* < 0.01 vs. LPS; ***, *p* < 0.001 vs. LPS. Statistical significances were checked with ANOVA and Tukey test.

**Table 4:** List of GenBank accession numbers used for PCR primers.

Gene Name	GenBank Accession Number
*Chop*	NM_007837
*Camk2α*	NM_012920
*Stat1*	NM_009283.4
*Stat3*	NM_011486.5
*Jak2*	NM_001048177.3
*Fas*	NM_007987
*c-Jun*	NM_010591
*c-Fos*	NM_010234
*Nos2*	NM_010927.3
*Ptgs2*	NM_011198
*TLR3*	NM_126166
*TLR9*	NM_031178.2
*β-Actin*	NM_007393.3

## Data Availability

The data presented in this study are available on request from the corresponding author.

## Supplementary Materials

The following supporting information can be downloaded at: https://www.mdpi.com/article/10.3390/ijms24032062/s1.
