# Supplementary material for: Moutan Cortex Extract Modulates Macrophage Activation via Lipopolysaccharide-Induced Calcium Signaling and ER Stress-CHOP Pathway
Source: Int J Mol Sci. 2023 Jan 20;24(3):2062. doi: 10.3390/ijms24032062 (PMC9916843; doi:10.3390/ijms24032062)
Supplement: Supplementary file 1 [file ijms-24-02062-s001.zip › ijms-2096458-supplementary.pdf]

< Supplementary Material for Materials and Methods>

1. Materials and Methods

The following provides a detailed description of the materials and experimental methods used in this study.

1.1. Materials

Dulbecco's modified Eagle's medium (DMEM), FBS, penicillin, streptomycin, phosphate-buffered saline (1x), lipopolysaccharide (LPS) from Escherichia coli O55:B5 (Cat No. L2880; endotoxin levels of not less than 500,000 EU), indomethacin (Cat No. 17378), baicalein (Cat No. 465119), rutin, and other cell culture reagents were purchased from Millipore (Billerica, MA, USA). Griess reagent assay kits and Fluo-4 calcium assay kits were purchased from Thermo Fisher Scientific (Waltham, MA, USA). Dihydrorhodamine 123 assay kits were purchased from Millipore. Multiplex cytokine assay kits were purchased from Millipore. Real time PCR kits were ordered from Bio-Rad (Hercules, CA, USA). Fixable Viability Dye eFluor 520 (eBioscience 65-0867-18, Life Technologies Corporation, Carlsbad, CA, USA), phospho-p38 MAPK (T180/Y182) Antibody (eBioscience 17-9078-42), phospho-STAT3 (Tyr705) Antibody (eBioscience 12-9033-42), phospho- IκB-α Antibody (Ser32, Ser36) (eBioscience 12-9035-42), and Mouse IgG2b kappa Isotype Control (eBioscience 12-4732-81) were obtained from Life Technologies Corporation (Carlsbad, CA, USA). All other solutions for flow cytometric analysis were purchased from Thermo Fisher Scientific.

1.2. Preparation of CP

Commercial Moutan Cortex (the cortex of *Paeonia suffruticosa* root; CP) was obtained from Omniherb (Daegu, Korea) and authenticated by Professor W. Park of Gachon University while referring to the website of the Ministry of Food and Drug Safety, Republic of Korea (https://www.nifds.go.kr/nhmi/analscase/snststMnl/view.do?selectedSnststMnlNo=134 or https://www.nifds.go.kr/nhmi/hbdc/ofcmhbdc/view.do?selectedDmstcOfcmNo=161&selectedMdntfNo=266). A voucher specimen (No. 21032) was deposited at the Department of Pathology in Gachon University's College of Korean Medicine. As herbal drugs are traditionally extracted using hot water, in the present study, Moutan Cortex was extracted with boiling water for 2 h, filtered with Advantec Grade No. 2 Filter Paper (5 µm), and then lyophilized (yield: 33.98%). The powdered extract (CP) (25~200 mg/mL) was dissolved in phosphate buffer saline (1x) aseptically, then filtered through a 0.22 µM syringe filter.

1.3. Total Flavonoid Content of CP

The total flavonoid content of CP was determined using the diethylene glycol colorimetric method. Briefly, the sample solution (20 µL of 2 mg/mL CP) was mixed with 200 µL of diethylene glycol and 20 µL of 1 N NaOH. The sample absorbance was read at 405 nm using TRIAD LT spectrofluorometer (Dynex, West Sussex, UK) after 1 h of incubation at 37°C. Rutin (Cat No. PHL89270) was used as a reference standard, and the total flavonoid content was expressed in terms of milligrams of rutin equivalents (mg RE/g extract).

1.4. Cell culture and cell viability

RAW 264.7 mouse macrophages were obtained from Korea Cell Line Bank (Seoul, Korea). RAW 264.7 mouse macrophages were cultured with DMEM supplemented with 10% FBS containing 100 U/mL of penicillin and 100 µg/mL of streptomycin at 37 °C in a 5% CO_2 humidified incubator. Prior to the experimental assays, RAW 264.7 mouse macrophages were washed with phosphate buffer saline (1x). Briefly, RAW 264.7 mouse macrophages were incubated with CP for 24 h in 96-well plates (1x10^4 cells/well) to verify the toxicity of CP. After 24 h culture with CP, cell viability was confirmed with the tetrazolium-based colorimetric assay (a modified MTT assay). OD was determined at 540 nm with a Model 680 microplate reader (Bio-Rad, Hercules, CA, USA).

1.5. Quantification of NO Production in RAW 264.7 mouse macrophages

The NO concentration in culture medium was determined by the Griess reagent assay. Specifically, after incubating RAW 264.7 mouse macrophages (1x10^4 cells/well) with LPS and/or CP for 24 h, 100 µL of supernatant from each well was mixed with 100 µL Griess reagent in a 96-well plate. After 15 min of incubation at room temperature, the optical density was determined at 540 nm with a Model 680 microplate reader (Bio-Rad, Hercules, CA, USA). Indomethacin (0.5 µM) was used as a positive control.

1.6. Ca^{2+} release in RAW 264.7 mouse macrophages

The Ca^{2+} release from RAW 264.7 mouse macrophages was measured using a Fluo-4 calcium assay kit (Thermo Fisher Scientific). RAW 264.7 mouse macrophages in 96-well plates ($1x10^5$ cells/well) were incubated with LPS and/or CP for 24 h at 37 °C. Next, the medium was removed, and the cells were incubated with 100 μL of the Fluo-4 dye loading solution for 30 min at 37 °C. After the incubation, the fluorescence intensity in each well was determined using TRIAD LT spectrofluorometer with excitation and emission filters of 485 nm and 535 nm, respectively.

1.7. Hydrogen peroxide production in RAW 264.7 mouse macrophages

Hydrogen peroxide production in RAW 264.7 mouse macrophages ($1x10^4$ cells/well) was measured by dihydrorhodamine 123 (DHR) assay. During the cellular production of reactive oxygen species, the nonfluorescent DHR was oxidized by H_2O_2 and irreversibly converted to the green fluorescent compound rhodamine 123 (R123). R123 was membrane-impermeable and accumulated in the cells. An aliquot of DHR (prepared to produce a concentration of 10 μM in each well) was added to each 96-well plate and pre-incubated for 30 min at 37 °C. Then, the medium was removed, and RAW 264.7 mouse macrophages were incubated with LPS and/or CP for 24 h, 18 h, and 72 h at 37 °C. After incubation, the fluorescence intensities of each well were analyzed by a spectrofluorometer (Dynex) with an excitation filter 485 nm and an emission filter 535 nm.

1.8. Multiplex Cytokine Assay for cytokines production in RAW 264.7 mouse macrophages

Cytokines from RAW 264.7 mouse macrophages after 24 h of treatment were evaluated with MILLIPLEX MAP Mouse Cytokine/Chemokine Magnetic Bead Panel kits (Millipore) and a Bio-Plex 200 suspension array system (Bio-Rad, Hercules, CA, USA). Briefly, RAW 264.7 mouse macrophages were seeded in 96-well plates ($1x10^4$ cells/well) and treated with LPS and/or CP [15]. After 24 h of treatment, the levels of the following cytokines in each well were analyzed: interleukin (IL)-6, IL-10, monocyte chemoattractant protein (MCP)-1, tumor necrosis factor (TNF)-α, leukemia inhibitory factor (LIF), lipopolysaccharide-induced CXC chemokine (LIX; CXCL5), granulocyte colony-stimulating factor (G-CSF), granulocyte macrophage colony-stimulating factor (GM-CSF), vascular endothelial growth factor (VEGF), macrophage colony-stimulating factor (M-CSF), macrophage inflammatory proteins (MIP)-1α, MIP-1β, MIP-2, and RANTES (CCL5; regulated on activation, normal T cell expressed and secreted). Cytokines were measured using a Luminex assay based on xMAP technology using MILLIPLEX MAP Mouse Cytokine/Chemokine Magnetic Bead Panel kits and a Bio-Plex 200 suspension array system. The assay conducted in this experiment was designed for the multiplexed quantitative measurement of multiple cytokines in a single well, and as little as 25 μL of cell culture supernatant was used. The standard curves for each cytokine were generated using the kit-supplied reference cytokine samples. Briefly, the following procedure was performed: after pre-wetting the 96-well plate with Wash Buffer, the Wash Buffer was removed from each well using a Handheld Magnetic Separation Block (HMSB). Next, cell culture supernatants from each well were incubated with antibody-conjugated beads on a plate shaker for 2 h at room temperature. After incubation, well contents were gently removed with a HMSB, and the 96-well plate was washed twice. Then, 25 μL of Detection Antibodies was added to each well and incubated with agitation on a plate shaker for 1 h at room temperature. Subsequently, 25 μL Streptavidin–Phycoerythrin was added to each well containing the Detection Antibodies and incubated for 30 min with agitation on a plate shaker at room temperature. After incubation, the well contents were gently removed and washed 2 times using a HMSB. Then, 150 μL of Sheath Fluid was added to all wells, and the beads bound to each cytokine were analyzed with a Bio-Plex 200 instrument (Bio-Rad). The raw data (fluorescence intensity) were analyzed using Bio-Plex Manager software (Bio-Rad). Baicalein (25 μM), a well-known anti-inflammatory flavonoid, was used as a positive control.

1.9. Quantitative Real time PCR for mRNA Expression in RAW 264.7 mouse macrophages

Total RNA of RAW 264.7 mouse macrophages ($1x10^6$ cells/well) was isolated using NucleoSpin RNA kit (Macherey-Nagel, Duren, Germany). Then, cDNA of the RNA samples was synthesized using iScript cDNA Synthesis kit (Bio-Rad) and checked with an Experion Automatic Electrophoresis System (Bio-Rad). The transcription *Chop*, *Camk2α*, *Stat1*, *Stat3*, *Jak2*, *Fas*, *c-Jun*, *c-Fos*, *Nos2*, *Ptgs2*, *TLR3*, *TLR9*, and *β-Actin* (internal control) was evaluated with Quantitative real time PCR using Bio-Rad CFX 96 (Bio-Rad). The details of the step-by-step experiment are as follows.

1.9.1. Isolation of RNA

RAW 264.7 mouse macrophages were incubated with LPS and/or CP for 18 h in 6-well plates ($1x10^6$ cells/well). Baicalein (25 μM), a well-known anti-inflammatory flavonoid, was used as a positive control. After 18 h of incubation, the total RNA in each well was isolated using NucleoSpin RNA kit (Macherey-Nagel, Duren, Germany). Briefly, 350 μL Lysis Buffer RA1 and 3.5 μL β-mercaptoethanol were added to the cell pellet and vigorously vortexed to lyse cells. Lysate was cleared by filtration using a NucleoSpin® Filter, then 350 μL ethanol (70%) was added, and mixed by vortexing.

The lysate was loaded into the NucleoSpin® RNA Column, and 350 µL Membrane Desalting Buffer was added and centrifuged. 95 µL DNase reaction mixture was applied directly to the center of the silica membrane of the column, followed by incubation at room temperature for 15 min. The samples were washed with Wash Buffer RA2 and Wash Buffer RA3, and silica membrane was dried. RNA was eluted in 60 µL RNase-free water and centrifuged.

1.9.2. Determination of RNA concentration

RNA concentration was measured using Experion RNA StdSens Analysis kit (Bio-Rad) with the Experion Automatic Electrophoresis System (Bio-Rad). First, the electrodes were cleaned using a cleaning chip filled with 900 µL DEPC-treated water. Then, the Gel-Stain solution was prepared, 9 µL of the solution was added into labeled wells, and the chip was primed. Samples and RNA ladder were loaded into the chip, which was vortexed using the Experion vortex station for 1 min. Lastly, the chip was loaded into the electrophoresis platform and the RNA StdSens Analysis program was run.

1.9.3. cDNA synthesis

cDNA of the RNA samples was produced using iScript cDNA Synthesis kit (Bio-Rad). Briefly, 20 µL complete reaction mixes were prepared using 5x iScript Reaction Mix (4 µL), iScript Reverse Transcriptase (1 µL), Nuclease-free water (variable), and RNA template (variable, 1 µg total RNA). The reaction mix (20 µL) was incubated in a thermal cycler (C1000 Thermal Cycler, Bio-Rad) according to the manufacturer's protocol (priming at 25°C for 5 min, reverse transcription at 46°C for 20 min, and RT inactivation at 95°C for 1 min).

1.9.4. Quantitative Real time PCR analysis

Gene expression was measured using quantitative polymerase chain reaction with iQ SYBR Green Supermix (Bio-Rad) using the CFX96 Real-Time PCR Detection System (Bio-Rad). Briefly, a master mix was prepared for all reactions by adding iQ SYBR Green Supermix and Forward/Reverse primers for each target gene. This master mix was thoroughly mixed to ensure homogeneity, and 7 µL was dispensed into the wells of a qPCR plate. Next, 3 µL of cDNA was added to each well; any air bubbles in the vessel bottom were removed, and the PCR plate was loaded into the real time PCR instrument. PCR was performed using the following protocol: denaturation of DNA at 95°C for 3 min, followed by 40 cycles of 95°C for 10 sec and 55°C for 30 sec. The $2^{-\Delta\Delta Ct}$ cycle threshold method was used to normalize the relative mRNA expression levels to the internal control, *β-actin*. Table S1 lists the primers used in this assay.

Table S1. Primers used in quantitative real time PCR

Name[1]	Forward Primer (5′–3′)	Reverse Primer (5′–3′)
Chop	CCACCACACCTGAAAGCAG	TCCTCATACCAGGCTTCCA
Camk2α	AGCCATCCTCACCACTAT	ATTCCTTCACGCCATCATT
Jak2	TTGGTTTTGAATTATGGTGTCTGT	TCCAAATTTTACAAATTCTTGAACC
Fas	CGCTGTTTTCCCTTGCTG	CCTTGAGTATGAACTCTTAACTGTGAG
Nos2	TGGAGGTTCTGGATGAGAGC	AATGTCCAGGAAGTAGGTGAGG
Ptgs2	TCAAACAGTTTCTCTACAACAACTCC	ACATTTCTTCCCCCAGCAA
Stat1	TGAGATGTCCCGGATAGTGG	CGCCAGAGAGAAATTCGTGT
Stat3	GTCTGCAGAGT TCAAGCACCT	TCCTCAGTCACGATCAAGGAG
c-Jun	ACTGGGTTGCGACCTGAC	CAATAGGCCGCTGCTCTC
c-Fos	AGAGCGGGAATGGTGAAGA	TCTTCCTCTTCAGGAGATAGCTG
TLR3	AAAGTTCTGGGAAAGTCTCTAAATGT	AACAAAAACCATAATCTGCATTGA
TLR9	GAATCCTCCATCTCCCAACAT	CCAGAGTCTCAGCCAGCACT
β-Actin	CTAAGGCCAACCGTGAAAAG	ACCAGAGGCATACAGGGACA

[1]Primer names: C/EBP homologous protein (*Chop*), calcium/calmodulin dependent protein kinase II alpha (*Camk2α*), Janus kinase 2 (*Jak2*), First apoptosis signal receptor (*Fas*), Nitric oxide synthase 2 (*Nos2*), Prostaglandin-endoperoxide synthase 2 (*Ptgs2*), Signal Transducer and Activator of Transcription 1 (*Stat1*), *Stat3*, *c-Jun*, *c-Fos*, *Toll-like receptor 3 (TLR3)*, *TLR9*, and *β-Actin*.

1.10. Flow Cytometric Analysis for the level of Phosphorylated STAT3, Phosphorylated p38 MAPK, and Phosphorylated IkB-α

RAW 264.7 mouse macrophages were incubated with LPS and/or CP for 18 h in 6-well plates (3 x 10^5 cells/well), and the levels of phosphorylated p38 MAPK, phosphorylated STAT3, and phosphorylated IkB-α in RAW 264.7 were evaluated via flow cytometry using an Attune NxT flow cytometer (Thermo Fisher Scientific). Briefly, after 18 h of treatment, cells were stained with Fixable Viability Dye eFluor 520 (eBioscience 65-0867-18), phospho-STAT3 (Tyr705) Antibody (eBioscience 12-9033-42), phospho-p38 MAPK (T180/Y182) Antibody (eBioscience 17-9078-42), phospho-IκB-α Antibody (Ser32, Ser36) (eBioscience 12-9035-42), and Mouse IgG2b kappa Isotype Control (eBioscience 12-4732-81) according to the manufacturer's protocol. Fixable Viability Dye eFluor 520 was used to irreversibly label dead cells prior to cryopreservation, fixation, and/or permeabilization procedures. Cells were fixed with Fix Buffer (Thermo Fisher Scientific), permeabilized with Perm Buffer (Thermo Fisher Scientific), and stained with fluorescent-labeled antibodies. The stained cells were analyzed on the Attune NxT flow cytometer (Thermo Fisher Scientific). A serial gating strategy used forward scatter versus side scatter plots, forward scatter versus viability stain plots, and the target antibody expression plots. Unstained cells were used as the negative controls for gating. The data were obtained from the mean fluorescent intensities of the samples. Details regarding startup, proper calibration, and operation of the Attune can be found in the Attune User Guide (https://assets.thermofisher.com/TFS-Assets/LSG/manuals/100024235_AttuneNxT_HW_UG.pdf).

1.11. Statistical analyses

Data are representative of at least three independent experiments, and the values are expressed in means ± standard deviation (SD). All data were analyzed by one-way analysis of variance (ANOVA) test followed by Tukey's multiple comparison test using GraphPad Prism 4.0 (GraphPad Software, San Diego, CA, USA).
